# A Study of Morbidity Pattern in Street Sweepers: A Cross-sectional Study

**DOI:** 10.4103/0970-0218.43226

**Published:** 2008-10

**Authors:** Yogesh D Sabde, Sanjay P Zodpey

**Affiliations:** Department of Preventive and Social Medicine, Government Medical College and Hospital, Nagpur, Maharashatra, India

**Keywords:** Cross-sectional study, occupational morbidities, street sweepers

## Abstract

**Background:**

Street sweepers play an important role in maintaining the health and hygiene within the cities. This job exposes the street sweepers to a variety of risk factors such as dust, toxins and diesel exhaust pollution, which make them vulnerable to develop certain occupational diseases. Therefore, it was thought necessary to study the morbidity profile in this occupational group.

**Objectives:**

To study the prevalence of morbidities among street sweepers and comparison group.

**Study Design:**

A cross-sectional study with a comparison group.

**Study Setting:**

Nagpur Municipal Corporation, Nagpur.

**Subjects:**

The study included two groups: (1) A study group comprising 273 street sweepers. (2) A comparison group comprising 142 class IV workers working in the office buildings of Nagpur Municipal Corporation, Nagpur.

**Materials and Methods:**

A pretested proforma was used to record the necessary information such as clinical history, sociodemographic factors, findings of clinical examination and investigations performed.

**Results and Conclusions:**

The important morbidities detected among street sweepers were the following: anemia (20.5%), hypertension (9.5%), upper respiratory tract infections (URTI) (7.3%) and chronic bronchitis (5.9%). In the comparison group, important morbidities detected were the following: anemia (20.4%), hypertension (11.3%), hyperacidity (9.9%), URTI (7.0%) and refractive error (7.0%). Chronic bronchitis was detected in two subjects (1.4%) of the comparison group. The prevalence of chronic bronchitis was significantly high among street sweepers than that of subjects of the comparison group. Therefore, it is recommended that further studies with a larger sample size be undertaken to identify the factors responsible for higher prevalence of chronic bronchitis among the street sweepers.

## Introduction

Street sweepers play an important role in maintaining the health and hygiene in the cities. This job exposes street sweepers to a variety of risk factors such as dust, bioaerosols, volatile organic matter and mechanical stress, which make them susceptible to certain occupational diseases.(1–3) The important morbid conditions detected in these workers include the diseases of the respiratory system and eye, accidents, injuries, cuts and wounds, skin infections, animal bites, etc.(4–7)

**Figure 1:**
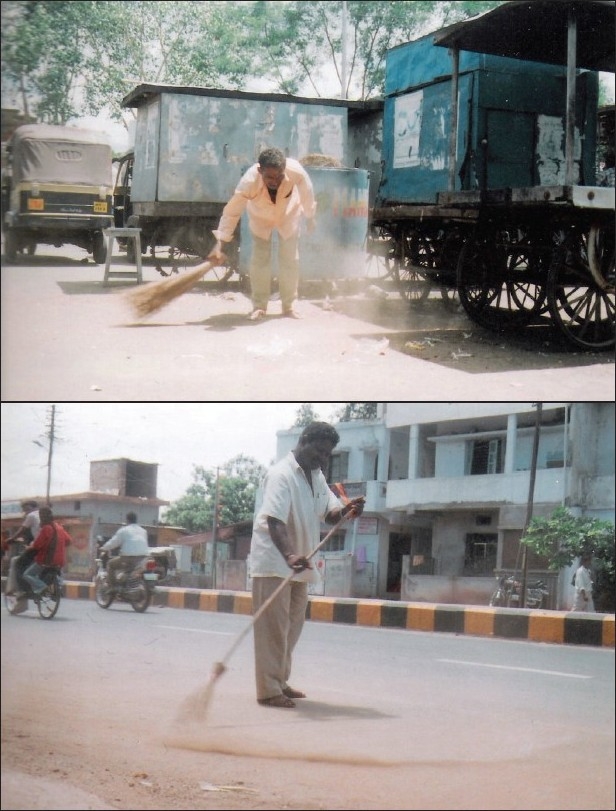
Street sweeper at work, not using any protective devices

**Figure 2:**
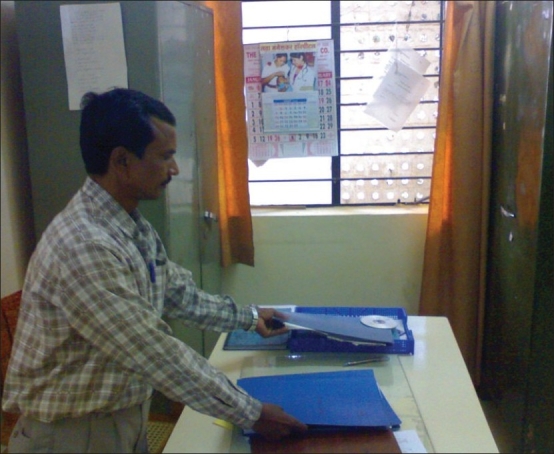
Class IV worker in office building

At present, the standards and norms for the management of municipal solid wastes in industrialized countries have substantially reduced the occupational health impacts. However, in developing countries, the health-related underpinnings of solid waste management still need to be addressed. Workers manually collect the wastes. There is little, if any, protection to workers from direct contact and injury and virtually no dust control at the workplaces. Solid waste collectors are exposed to significantly large amount of dusts, microorganisms, toxins and diesel exhaust pollution than the recommended norms [Figures 1 and 2].(8)

In India, the traditional culture has stigmatized street sweeping as a filthy and lowly occupation.(6) The medical problems of these workers are further compounded by various socioeconomic factors such as poverty, lack of education, poor housing conditions and poor diet.(4–7) Similarly, very few studies have been carried out in India to study the morbidity profile of these workers. With this background, and fortified by the fact that no such study has been carried out in Central India, we carried out the present research to study the prevalence of various morbidities in this occupational group.

## Materials and Methods

The present study was designed as a cross-sectional study with a comparison group. The study included two groups. The study group comprised all street sweepers working in a randomly selected zone (Hanumannagar Zone) of Nagpur Municipal Corporation, Nagpur; the comparison group comprised all class IV workers working in the office buildings of Nagpur Municipal Corporation, Nagpur.

We prepared a list of all study subjects. Group meetings of the subjects were arranged to apprise them of the purpose of the study and to ensure their cooperation. They were assured of the confidentiality of the data and their informed consent was obtained. A pretested proforma was employed to record the necessary information such as sociodemographic factors, occupational history, past and present medical history, findings of clinical examination and investigations performed among the workers. Standard clinical methods and investigations were used for the diagnoses of diseases, and opinion of the specialists from the Government Medical College, Nagpur, was obtained to confirm them. International Classification of Diseases version 10 (ICD 10) was used to perform the final diagnoses, e.g., Chronic bronchitis (ICD No. J44) was defined as the presence of a chronic productive cough in a patient on most of the days for three months and persistent chronic productive cough in a patient in for two successive years; and patients in whom other causes of chronic cough have been excluded (other causes of chronic cough were excluded by sputum microscopy and chest X-ray). To detect anemia in all the subjects, the hemoglobin level (g/dl) was estimated using Sahali's Hemoglobinometer. The subjects in whom the hemoglobin values were lower than that prescribed by the WHO norms were diagnosed with anemia (males < 13 g/dl and females < 12 g/dl). Injuries that had occurred in the past six months were included in the study.

During the personal interview, the participants were asked specific questions regarding the provision of personal protective devices (e.g., face masks, goggles and shoes) and their actual use. Their answers were verified from the official records.

Motivation: During the personal interview, an enquiry was made as to whether the NMC organized any health education sessions for the workers. Similarly, they were asked whether the medical officers or senior staff of NMC give any personal advice to the workers regarding the use of personal protective devices and quitting habits such as smoking and tobacco chewing.

Test for the statistical significance was applied by using x^2^ test for analyzing the difference between the two proportions (*P* < 0.05 was considered significant).

## Results

The present study comprised 273 street sweepers and 142 class IV employees (comparison group) working in Nagpur Municipal Corporation, Nagpur.

Table 1 shows the distribution of study subjects according to age and gender. A majority of the subjects belonged to the age group of 30 to 50 years. The mean age of the street sweepers was 39.70 years (S.D. = 7.15) and that of the comparison group was 41.99 years (S.D. = 8.40). The percentage of males was more than 50% in both the groups.

**Table 1:** Distribution of subjects according to age and gender

Age and gender	Street sweepers		Comparison group	
		
	No.	%	No.	%
**Age group (years)**				
20–29	19	7.0	13	9.2
30–39	104	38.1	35	24.6
40–49	114	41.8	69	48.6
50–54	36	13.2	25	17.6
**Gender**				
Male	149	54.6	97	68.3
Female	124	45.4	45	31.7

Table 2 shows that among the street sweepers, the morbid conditions commonly affected respiratory system (15%), followed by cardiovascular system (9.9%) and eyes (9.2%). In the comparison group, the morbid conditions commonly affected the cardiovascular system (12.7%), followed by eyes (9.9%) and respiratory system (9.2%). The prevalence of morbidities of other systems was similar in both the groups.

**Table 2:** System-wise distribution of morbid conditions among subjects

Morbid conditions	Street sweepers n = 273		Comparison group n = 142	
		
	No.	%	No.	%
Respiratory system	41	15.0	13	9.2
Cardiovascular system	27	9.9	18	12.7
Endocrine system	3	1.1	2	1.4
CNS	2	0.7	0	0.0
Gastrointestinal system	3	1.1	1	0.7
Musculoskeletal system	8	2.9	4	2.8
Eye	25	9.2	14	9.9
Skin	4	1.5	3	2.1
Injuries	3	1.1	0	0.0

Table 3 shows the morbid conditions according to ICD 10 System of Disease Classification. It was observed that the important morbid conditions affecting the respiratory system of the street sweepers were upper respiratory tract infections (URTI) in 7.3%, followed by chronic bronchitis (5.9%) and bronchial asthma (1.8%). In the comparison group, URTI was detected among 7.0% of the subjects, followed by chronic bronchitis among 1.4% and bronchial asthma among 0.7% subjects. The prevalence of chronic bronchitis was significantly high among street sweepers than that in the comparison group (*P* < 0.05). The prevalence of hypertension was 9.5% among street sweepers, and 11.3% among comparison group. Morbid conditions affecting the eyes among the street sweepers were refractive error (3.7%), pterygium (2.9%) and acute atopic conjunctivitis (2.6%), whereas in the comparison group 7.0% had refractive error followed by pterygium and corneal opacity (1.4% each). Anemia was noted in 20.5% street sweepers and 20.4% subjects in the comparison group.

**Table 3:** Distribution of morbid conditions among study subjects

ICD code	Morbid condition	Street sweepers n = 273		Comparison group n = 142	
			
		No.	%	No.	%
J41	Chronic bronchitis	16	5.9	2	1.4
J45	Bronchial asthma	5	1.8	1	0.7
J00	URTI	20	7.3	10	7.0
J49	Bronchiectasis	1	0.4	0	0.0
I10	Hypertension	26	9.5	16	11.3
I20.8	IHD	1	0.4	3	2.1
E14	Diabetes mellitus	3	1.1	2	1.4
G81	Hemiparesis	1	0.4	0	0.0
G83	Facial palsy	1	0.4	0	0.0
I84	Hemorrhoids	3	1.1	1	0.7
M43.2	Cervical spondylosis	2	0.7	1	0.7
M43.6	Lumbar spondylosis	1	0.4	0	0.0
M15.4	Arthritis knees	6	2.2	3	2.1
H11.4	Pterygium	8	2.9	2	1.4
H11.9	Acute atopic conjunctivitis	7	2.6	1	0.7
H17	Corneal opacity	2	0.7	2	1.4
H52.7	Refractive error	10	3.7	10	7.0
L23	Contact dermatitis	4	1.5	3	2.1
S50	Superficial injury on forearm	1	0.4	0	0.0
S80	Superfiscial injury on lower leg	2	0.7	0	0.0
D50	Anemia	56	20.5	29	20.4
K02.9	Dental caries	8	2.9	6	4.2
K25	Hyperacidity*	10	3.7	14	9.9
K30	Constipation*	1	0.4	3	2.1
R29.8	Backache*	12	4.4	5	3.5

* Symptoms

No fatal accident was recorded in the past six months among these workers.

In this study, none of the 273 street sweepers were noted to be using protective devices such as face mask, goggles, gumboots or gloves while working. The reasons given were an irregular supply of the protective devices and lack of motivation for using these devices [Figure 1].

The percentage of smokers was higher among street sweepers (17.90%) than that among subjects in the comparison group (10.46%). Smoking is a known risk factor causing chronic bronchitis that is an important morbidity condition among street sweepers.

## Discussion

The results of this study revealed that the prevalence of chronic bronchitis was significantly high among street sweepers (5.9%) than that among subjects of the comparison group. The high prevalence of chronic bronchitis could be attributed to occupational exposure to dust and smoking habits among street sweepers. Other important morbidities include bronchial asthma, pterygium and conjunctivitis.

These findings are in agreement with those of the study conducted among waste collectors in Denmark,(9) where the prevalence of chronic bronchitis (7.8%) was significantly higher than that among park workers.

Raaschou-Nielsen O *et al.*(10) also found a significantly higher prevalence of chronic bronchitis and asthma in street cleaners of Copenhagen than in cemetery workers.

Diggikar UA(4) studied the morbidity profile of female street sweepers working in Pimpri-Chinchwad Municipal Corporation (PCMC), Pune. The overall morbidity revealed anemia in 89.1%, backache in 16.2%, recurrent respiratory infection in 10.2%, hypertension in 5.1% and skin allergy in 4.2% of the female sweepers. The prevalence of recurrent respiratory infection was significantly higher among sweepers than in the subjects of the control group.

Nagraj C *et al.*(5) studied the morbidity profile of sweepers working under Bangalore City Corporation. The major ailments encountered were hypertension (18.9%), respiratory ailments (7.78%) and skin ailments (3.11%).

In all the above mentioned studies, similar age groups were used as the age for recruitment and retirement of the workers in any Municipal Corporation.

A study by Gupta SC(6) in 1962 on the street sweepers and their families in Lucknow revealed that the most frequently detected morbidities were skin diseases (2.6%), URTI (0.8%), bronchitis (0.5%) and conjunctivitis (0.35%). It should be noted that the prevalence rate of these morbidities in this study was considerably lower than that observed in the recent studies. This could be because of the inclusion of the family members, which might have diluted the effect of occupational exposure. However, it can also be assumed that rapid urbanization over past few decades led to an increased occupational exposure and thus increased occupational morbidity among these workers.

In this study, it was revealed that NMC runs dispensaries for these workers, which provide free treatment for the diseases. However, no preplacement or any regular health examinations for these workers are held at these dispensaries. No health education sessions are arranged by NMC for these workers; further, the medical officers also do not give any personal advice to the workers regarding the use of personal protective devices or quitting habits such as smoking.

## Conclusion

The important morbidities detected among the street sweepers were chronic bronchitis, bronchial asthma, pterygium and conjunctivitis. The problem of chronic bronchitis was observed to be significant among these workers, which might be attributable to the occupational exposure to dust and smoking habits among these workers. These findings are in agreement with those of other studies conducted on these workers. Based on these findings, we recommend the following:

1. Further studies with larger sample size should be undertaken to identify the factors responsible for increased prevalence of chronic bronchitis among street sweepers.
2. The use of protective devices such as masks and goggles by these workers should be promoted in order to reduce the occupational exposure to dust.
3. The workers should be motivated to quit smoking.
4. Occupational health services should be provided to these workers, which should include regular health examination.
5. Automatization of street sweeping should be considered to reduce occupational exposure to dust.
